# A digital yoga-based ‘Pause Grow Reflect’ intervention for postgraduate researchers: a feasibility randomised controlled trial and embedded qualitative acceptability study

**DOI:** 10.1186/s40814-026-01807-9

**Published:** 2026-06-12

**Authors:** Natalie Bisal, Riya Patel, Nikki Holliday, Deborah Lycett, Andy Turner, Maxine Whelan

**Affiliations:** 1 Centre for Healthcare and Community Transformation, Coventry University, Coventry, UK; 2 University of Birmingham, Birmingham, UK; 3 Centre for Ethnic Health Research, NIHR Applied Research Collaboration-East Midlands (ARC-EM), University of Leicester, Leicester, UK

**Keywords:** Digital health, Feasibility, Acceptability, Postgraduate students, Mental well-being, Self-care, Yoga

## Abstract

**Background:**

The prevalence of mental health and well-being problems among postgraduate researchers (PGRs) is concerning. Self-care is a protective factor; however, engagement can be low. Interventions to date are not theory-driven nor co-developed. This study assessed the feasibility and acceptability of a co-created, theory-driven digital yoga-based self-care intervention (Pause Grow Reflect).

**Methods:**

A 2:1 randomised feasibility trial of the 7-week Pause Grow Reflect intervention and embedded qualitative study was conducted in UK PGRs. Primary outcomes included rates of recruitment, retention, follow-up, adherence, and engagement. Secondary outcomes assessed fidelity, acceptability, adverse events, and preliminary signals of efficacy. Criteria determined progression to a definitive trial. An embedded qualitative study comprising 1:1 semi-structured interviews was guided by the Theoretical Framework of Acceptability (TFA) and data analysed using deductive thematic analysis.

**Results:**

PGRs were allocated to the intervention (*n* = 24) or control (*n* = 13) group. Progression criteria were met for recruitment, T1 assessments, and acceptability, whilst rates of retention, engagement, and T2 follow-up assessments were not. Marginal mean differences for stress were observed. The qualitative data (*n* = 10 interviews) showed that all seven TFA domains were important, with issues mostly reported for the constructs of ‘burden’ and ‘opportunity costs’.

**Conclusion:**

Pause Grow Reflect was largely feasible to deliver and acceptable within a UK context, and may be a promising offer to promote self-care during doctoral programmes. Further intervention refinement would promote better rates of retention, adherence, and engagement. Preliminary observations were positive, though a larger RCT is needed to determine efficacy.

**Supplementary Information:**

The online version contains supplementary material available at 10.1186/s40814-026-01807-9.

## Key messages regarding feasibility

- What uncertainties existed regarding the feasibility?
  We had uncertainties about whether postgraduate students would like and engage with a digital yoga-based self-care intervention. We had specific uncertainties around study recruitment, retention, and follow-up rates, as well as adherence and engagement rates through the intervention. Acceptability was another key aspect to this study, to understand postgraduate researchers’ views of the intervention.
- What are the key feasibility findings?
  Nearly four in five people were considered eligible for the study and, of whom, 82% consented to take part. Retention across the whole sample was 32.4% with follow-up rates at 81% at timepoint 1 (6 weeks). Levels of engagement measured via mean percentage of pages accessed decreased as the intervention progressed. Most (83%) participants viewed the intervention as acceptable but 40% of participants described how engaging in Pause Grow Reflect interfered with other priorities. Six negative unintended effects were reported during or directly after yoga sessions.
- What are the implications of the feasibility findings for the design of the main study?
  Changes to the Pause Grow Reflect intervention should be implemented prior to a larger scale study being conducted. Our results indicate that recruitment of postgraduate researchers is feasible, but we will likely encounter challenges with retention. The qualitative findings have been valuable in understanding some of the key domains of acceptability where Pause Grow Reflect is acceptable and less acceptable to this population. Our findings suggest that the selected outcome measures captured meaningful outcome trends, encouraging their use in the next study.

## Introduction

The process of completing doctoral studies can be challenging and unpredictable for postgraduate researchers [1], both in terms of progress and the extent to which emotions and capabilities impact stress levels and well-being [2, 3]. This transition is often accompanied by uncertainty [3–5] and feelings of unpreparedness, along with several psychological, intellectual, and social changes [2, 6–8]. These circumstances place heavy demands, requiring students to acquire new knowledge and skills whilst maintaining motivation and productivity [3].

Poor mental health and well-being is a critical problem facing postgraduate researchers, with greater levels of stress noted compared to the general population [9]. Alarmingly, one in four UK postgraduate researchers report poor mental well-being [10]; these rates are consistent with doctoral students in Belgium [11], USA [12], and France [13] demonstrating how widespread the issue is. Rates of poor mental health and well-being, suicide, and the demand for mental health support and counselling have increased over recent years [14–16]. High levels of stress and psychological distress [11, 12, 17], and anxiety and depression [18] have also been widely reported. This can lead to reduced motivation, less productivity, lower academic satisfaction, and poor academic performance [19]. The COVID-19 pandemic has also further increased the risk of mental health problems among university students [20, 21]. The mental health and well-being status of university students in the UK is therefore an urgent priority to address.

Likely a contributing factor to poor mental well-being, postgraduate researchers have been reported as not taking time away from their studies to take care of themselves [3, 22, 23]. Self-care is understood as the practice of taking action to maintain or enhance health and well-being in a way that is personal to the individual [24]. Self-care has been found to be a protective factor for postgraduate researcher well-being [9]. A lack of self-care can be detrimental to health and well-being, life satisfaction, productivity, and overall PhD progress [9, 25]. Prioritising time for self-care is considered a challenge often influenced by workload and concerns around productivity and progress in studies [22]. Yet engaging in self-care activities may afford postgraduate researchers time away from their studies to nurture their identities outside of the PhD [9] and has been positively associated with well-being outcomes [26]. Higher education institutions have a responsibility to provide appropriate well-being support [27], yet there has been an over-reliance on traditional forms of support such as counselling that are not tailored to the specific needs of PGRs [14]. Postgraduate researchers have reported receiving minimal support towards understanding and managing their personal well-being during their doctoral training programme and so view their personal well-being as their own responsibility [28]. With postgraduate researchers less likely to disclose mental health problems and seek mental health and well-being support [14], there is a need for accessible support options. Digital support can offer user anonymity, greater accessibility, lower costs, and a low access threshold [29], as well as offer the potential for high scalability and wider implementation which universities would demand [30]. As intervention studies are lacking in postgraduate researcher populations [31], studies exploring interventions aimed at promoting self-care and mental well-being and those delivering support through digital methods are warranted.

Observational evidence suggests that holistic interventions might be beneficial for a range of mental health and well-being outcomes in university student populations. A review of self-care interventions found interventions comprising elements of psychoeducation, self-care techniques (e.g. breathing or meditation techniques), and reflection activities were effective in reducing stress among health science postgraduate students [32]. Similarly, yoga was identified as a holistic self-care strategy that offers multiple behavioural tools and practices along with a range of physical, emotional, and mental health and well-being benefits [33, 34]. Research also indicates the protective role of social support, sense of belonging, and academic community on student mental health and well-being [22, 35, 36]. Most studies exploring holistic self-care interventions have tended to focus on students from specific disciplines (e.g. healthcare, psychology, medicine); however, research supports that mind-body self-care interventions may offer a promising approach to supporting the self-care and well-being needs of postgraduate researchers more generally (e.g. [32, 34, 37]).

With co-created interventions more likely to result in appropriate, appealing, and feasible interventions for target users compared to those solely based on researcher-led top-down methods [38], postgraduate researchers were involved in the co-creation of a digital, self-care intervention called Pause Grow Reflect (Bisal et al., *under review*). As outlined by the MRC framework [39, 40], feasibility and acceptability testing is an important phase of the intervention development and evaluation process. This study aimed to assess the feasibility of Pause Grow Reflect using indicators of eligibility, recruitment, retention, follow-up, adherence and engagement, and acceptability.

## Methods

The CONSORT extension for feasibility and pilot studies [41] and COREQ [42] were used to inform the study write-up.

### Trial design, setting, and ethics approvals

This study was a feasibility trial using a randomised waitlist control group design with a 2:1 allocation ratio in the UK. An embedded qualitative study was also used comprising 1:1 semi-structured interviews. Ethics approvals were acquired from Coventry University’s Research Ethics Committee (ref #P151020).

### Participants

Postgraduate researchers (doctoral/PhD level) aged 18 years or older, enrolled at a UK-based university, able to proficiently read, write, speak, and understand English, and had access to the internet were eligible to participate. The Physical Activity Readiness Questionnaire for Everyone (PAR-Q+) was used to screen for eligibility [43]. Any individuals deemed ‘potentially eligible’ (using PAR-Q+) were contacted to confirm their responses. Pregnant postgraduate researchers were excluded due to ethical and safety considerations. It is recommended that, to include people who are pregnant in yoga intervention trials, specific adaptations should be made according to the trimester of their pregnancy. NB (intervention facilitator) did not hold the necessary qualifications to do this, and without this it was not deemed appropriate to include pregnant women as this was a self-guided intervention. This exclusion reflects a methodological precaution as prenatal yoga is safe only when specifically tailored and women are medically cleared to participate to minimise the occurrence of potential risk and safety concerns.

Recruitment took place from June to July 2023. Study adverts were sent internally at a UK university and externally via social media. Potential participants were directed to a screening questionnaire hosted on Qualtrics. Eligible postgraduate researchers then completed the consent form and baseline (T0) questionnaires. Data were collected on the recruitment process, including recruitment responses and rates.

### Randomisation

All participants screened as eligible and upon completing the T0 measures were randomly assigned with a 2:1 allocation ratio to the intervention or control group. Sequence generation was implemented using the randomisation feature in Qualtrics. Participant blinding was not possible as participants were informed about group allocation. Researcher blinding was also not possible as the lead researcher (NB) was a facilitator for the intervention.

### Intervention group

**Fig. 1:**
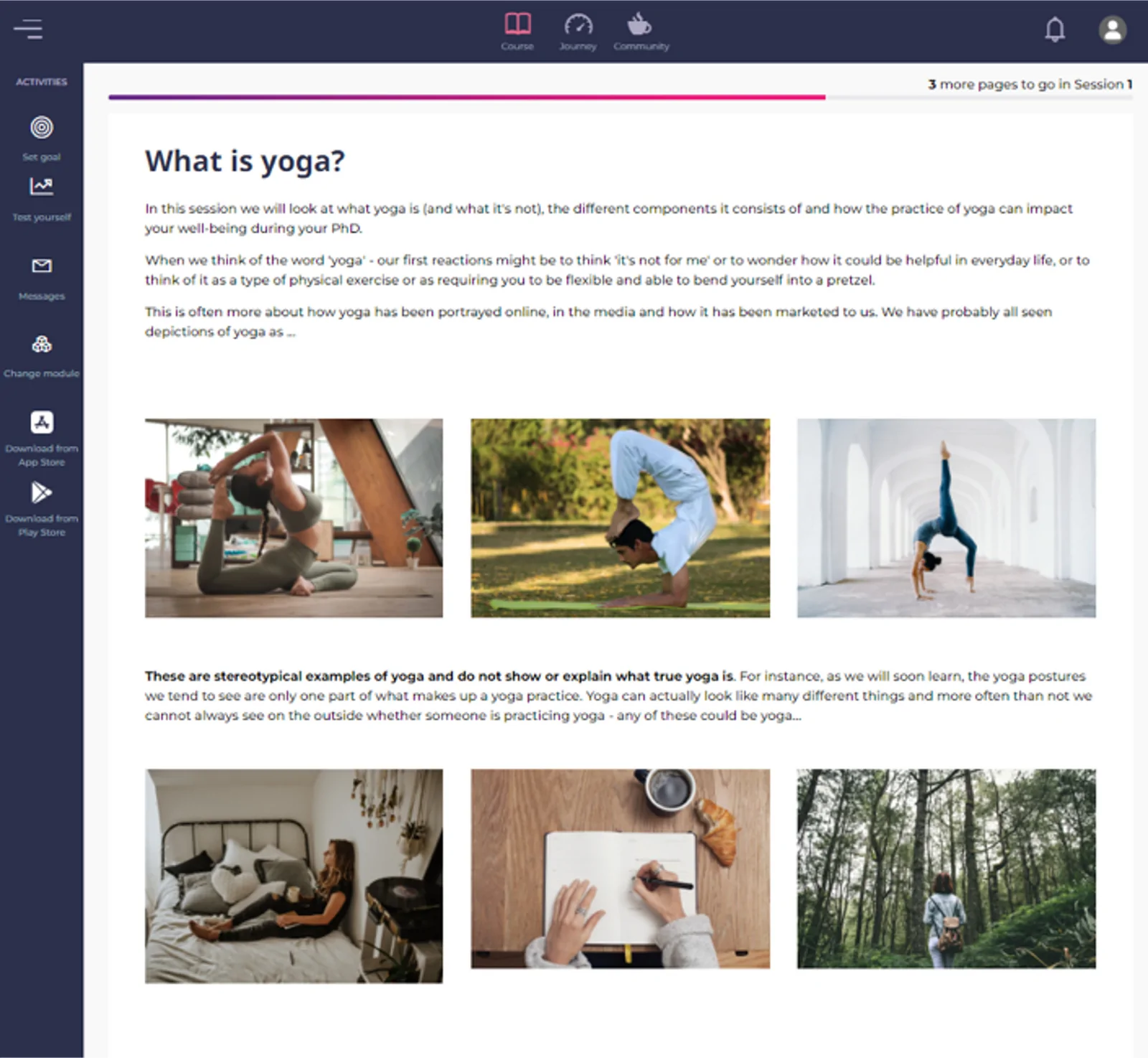
Example screenshots of the intervention

The intervention group received the Pause Grow Reflect programme from July to September 2023 (Fig. 1). This was a brief, online skills-based programme that incorporated principles and practices of yoga, self-care, stress management, and self-compassion. The development of Pause Grow Reflect has been previously reported (Bisal et al., *under review*) and is briefly outlined below.

The Pause Grow Reflect programme consists of six weekly sessions which guide participants through different topics to understand and identify their own self-care needs and preferences, providing the opportunity to try a range of yoga-based self-care tools and practices. Each session focused on a specific topic of the kosha model: physical, energetic, mental and emotional, wisdom and intellectual, and spiritual. The six sessions followed a similar format each week and consisted of psychoeducational content, behavioural (e.g. goal setting and feedback) and yoga-based activities (e.g. gratitude entry and self-reflection on yoga principles, yamas and niyamas), a social community space (including online forum and discussion topics), and pre-recorded videos guiding 30–40-min yoga practice that incorporate elements of breathing techniques, yoga postures (including provision of modifications), and guided meditation or mindfulness practice (Table 1; more detail provided in Supplementary Material 1). The classes were taught by the primary researcher (NB), an accredited and experienced yoga teacher. The intervention was facilitated by NB who supported participants throughout the programme and encouraged self-reflection and self-compassion. Access to the intervention included an additional, non-facilitated week at the end of the 6 weeks for participants to revisit or catch up on content. The intervention is described in greater detail according to the TIDieR checklist ([44]; Supplementary Material 2).

**Table 1:** An outline of the topics covered in the intervention sessions

Session	Example topics
Pre-session: Pause Grow Reflect introduction Session 1: Understanding self-care and yoga Session 2: The Physical Layer of experience Session 3: The Energetic Layer of experience Session 4: The Mental and Emotional Layer of experience Session 5: The Wisdom and Intellectual Layer of experience Session 6: The Spiritual (or Bliss) Layer of experience	• Introduction to content, platform, facilitator, and community • Self-care, well-being, yoga, and the koshas as a holistic self-care practice • Physical self-care (e.g. basic human needs), understanding stress • Breathing practices, signs of stress, and stressors of PGRs • Aspects of the mind, notion of awareness and mindfulness of thoughts, emotions, and beliefs • Concepts of the observing mind, discernment and concentration, and values • Connection (to self, others, nature, something greater), contentment, and joy

Content was released on a weekly basis. Pause Grow Reflect was hosted on the ‘Hope 4 The Community’ (H4C) platform which has been previously used in digital self-management interventions [45, 46]. An automated email was sent to participants informing them of the addition of new content. NB checked in with participants who had not engaged in the programme for two consecutive weeks.

### Control group

The control group were asked to continue typical behaviours before gaining access to Pause Grow Reflect at T1 (acting as a waitlist control). During the control period, the control group had no access to Pause Grow Reflect until after they completed post outcome measures.

### Sample size

Given the aims and scope of a feasibility trial, a sample size ranging from 10 to 20 participants per arm was considered sufficient, with no formal power calculation required [47]. A sample size of between 30 and 45 participants overall was considered sufficient.

### Outcomes

Several outcomes were measured. Where applicable, control group participants were assessed at the same time points (apart from T2) with an additional post-intervention point.

### Primary outcome: feasibility

#### Recruitment rates

Recruitment rates for participation and randomisation were recorded using Microsoft Excel and Qualtrics. Data were collected to capture how many individuals (a) expressed an interest, (b) screened for their eligibility, (c) provided informed consent, and (d) completed T0.

#### Retention and follow-up rates

Retention was measured based on number of sessions ≥75% attended. Follow-up rate was calculated by identifying participants who completed the study questionnaires at post-intervention (T1) and 3-month follow-up (T2; intervention only). An additional timepoint was embedded for post-intervention control group participants. Completion rates were based on participants who attended ≥4 sessions and completed T1 and T2.

#### Adherence and engagement rates

Adherence to the intervention sessions and content was recorded by the online platform. Patterns of engagement included the number of pages accessed in each session and the number of activities completed (e.g. goals set).

### Secondary outcomes

#### Differential attrition and representativeness

Differential attrition was based on whether substantial differences in attrition rates were observed between the two groups. Representativeness was measured based on whether differences were observed for (a) intervention compared to control and (b) T1 and T2 responders compared to non-responders.

#### Scores on outcome measures

Sociodemographic and academic characteristics and measures assessing yoga experience were collected at baseline only. The remaining outcome measures were assessed at baseline (T0), post-intervention (T1), and 3-month follow-up (T2; intervention only). All questionnaires were completed using Qualtrics. Two reminder emails were sent to participants within 1 week of the survey closing deadline.

#### Sociodemographic and academic characteristics

Data were collected for age, gender, ethnic background, sexual orientation, marital status, religion, faith or belief, caring responsibilities, employment status (alongside studies), parental education level, and disability, illness, or health condition. Academic characteristics included university institute enrolled at, fee status, mode, year, and subject discipline area.

#### Previous yoga or yoga-related experience

Participants were asked about previous experience of yoga or yoga-related activities. Questions determined the level of experience by ever practising yoga or yoga-related activities, regularity over the preceding 3 months (days/week and minutes/session) and current practice.

#### Yoga or yoga-related practice during the study

At T1 and T2, participants were asked whether they had engaged in yoga or yoga-related activities since the last data collection point. If participants answered ‘yes’, they reported the average number of days/week and minutes/session.

#### Self-care

Self-care behaviour was measured using the Mindful Self-Care Scale Brief version (B-MSCS; [48]) which covers the frequency of self-care behaviours across six domains: mindful relaxation, physical care, self-compassion and purpose, supportive relationships, supportive structure, and mindful awareness. The B-MSCS is a 24-item scale measured on a scale from 1 (never) to 5 (regularly: 6–7 days) over the past 7 days. Higher scores indicated greater frequency of self-care behaviour. Items were adapted, replacing ‘school life’, ‘schoolwork’, and ‘school tasks’ with ‘PhD life’, ‘PhD work’, and ‘PhD tasks’, respectively.

#### Mental well-being

Mental well-being was measured using the Short Warwick-Edinburgh Mental Well-Being Scale (SWEMWBS) [49] that covers thoughts and feelings relating to predominantly psychological and eudaimonic aspects of mental well-being. The 7-item SWEMWBS is measured on a scale ranging from 1 (none of the time) to 5 (all the time) with higher scores indicating greater positive mental well-being.

#### Perceived stress

Perceived stress was measured using the Perceived Stress Scale (PSS-10) [50] which includes statements about feelings and thoughts related to situations that are considered stressful. The scale uses a 10-item list rated on a 5-point scale ranging from 0 (never) to 4 (very often) with higher scores indicating greater levels of perceived stress.

#### Self-compassion

Self-compassion was measured using the Self-Compassion Scale Short Form (SCS-SF) which measures how participants act towards themselves during difficult times [51]. The scale is measured from 1 (almost never) to 5 (almost always). Higher overall scores indicated higher levels of self-compassion during difficult times.

#### Emotion regulation

Emotional regulation was measured using the Emotion Regulation Questionnaire (ERQ) [52] that measures individual differences in the use of emotion regulation strategies: cognitive reappraisal and expressive suppression. The 10-item ERQ is measured on a scale from 1 (strongly disagree) to 7 (strongly agree) with higher scores indicating higher usage of the cognitive reappraisal or expressive suppression strategies.

#### Mindful awareness

Mindful awareness was measured using the Mindful Attention Awareness Scale (MAAS) [53], which is a 15-item scale measuring attention to and awareness of present-moment experience in daily life. The scale is measured from 1 (almost always) to 6 (almost never) with higher scores reflecting higher levels of dispositional mindfulness.

#### Resilience

Resilience was measured using the Brief Resilience Scale (BRS) [54] which is a 6-item scale measuring the ability to bounce back or recover from stress. The scale is measured from 1 (strongly disagree) to 5 (strongly agree), with higher scores indicating greater levels of resilience.

### Intervention acceptability: quantitative

Retrospective participant acceptability was assessed using the Theoretical Framework of Acceptability questionnaire (TFA; [55]), with an open-text question to explore participant views on intervention acceptability.

### Intervention acceptability: qualitative

One-to-one, semi-structured interviews were conducted using Microsoft Teams within 2 weeks post-intervention. Participants were incentivised with a £10 voucher. Audio consent was recorded in a separate audio file before the interviews started, using Audacity software (3.5.0) and an external, digital Dictaphone application. The interview schedule (see Supplementary Material 3) was developed based on the Theoretical Framework of Acceptability (TFA) [55]. Participants could withdraw before, during, or up until 2 weeks after their interview.

### Intervention fidelity

The yoga sessions were pre-recorded. Automated emails and in-programme notifications were to be sent to participants on a Monday. As facilitator, NB was to offer encouragement to participants, stimulate discussion in the social community spaces, and respond to/like participants’ comments, goal setting, and feedback within 2 days. NB monitored for participant safety and responded to direct messages. In week 3 and week 5, NB checked in with participants who had not engaged for two consecutive weeks.

### Adverse event monitoring

Participants were directly asked to self-report any adverse events using an open-text question embedded within the programme.

### Deviations from protocol

Control group participants were invited to complete the additional post-intervention measures 1 week after the 6-week period (7 weeks after gaining access to the intervention) rather than after 6 weeks. Also, technical issues resulted in the week one practical yoga session being made available a day later than planned.

### Progression criteria

This study used criteria employed by other digital interventions delivered via the same online platform [45, 46]:

- Recruitment: ≥70% of eligible participants consented.
- Follow-up: ≥70% of participants completed T1 and T2 questionnaires.
- Retention: ≥50% of participants attended all six sessions.
- Engagement: ≥75% of participants viewed ≥75% of content in ≥3 sessions.
- Acceptability: ≥80% of participants viewed the programme as acceptable.

### Data analysis: quantitative

Data were collected in Qualtrics and analysed in Jamovi version 2.2.5. Frequencies and percentages (categorical data) and means and standard deviations (continuous data) were summarised overall and by group. Descriptive statistics were calculated for outcome measures at T0, T1, and T2 (latter for intervention group only). Where follow-up data could not be collected, intention-to-treat (ITT) analysis was conducted with missing data handled using a baseline-observation-carried-forward (BOCF) approach.

### Data analysis: qualitative

A deductive thematic analysis approach [56, 57] was used to organise the data within the seven TFA constructs [55] whereby data were analysed on a semantic level, aligning with a realist approach. Interviews were transcribed verbatim with potential identifiers excluded. NB initially read and re-read the transcripts to familiarise herself. The TFA was then used as a coding scheme to categorise data under the constructs. Data were then coded line-by-line before a list of codes was generated and collated. Codes were then grouped under the TFA themes and sub-themes identified. Discussions with the wider team (RP, MW) enabled peer review of the data interpretation and supporting extracts enhancing the credibility of the analysis. Discrepancies were discussed to ensure interpretations and findings were collectively agreed. If data did not reflect the TFA categories, data were analysed inductively. Findings are presented in line with the TFA with accompanying quotes.

## Results

### Recruitment

**Fig. 2:**
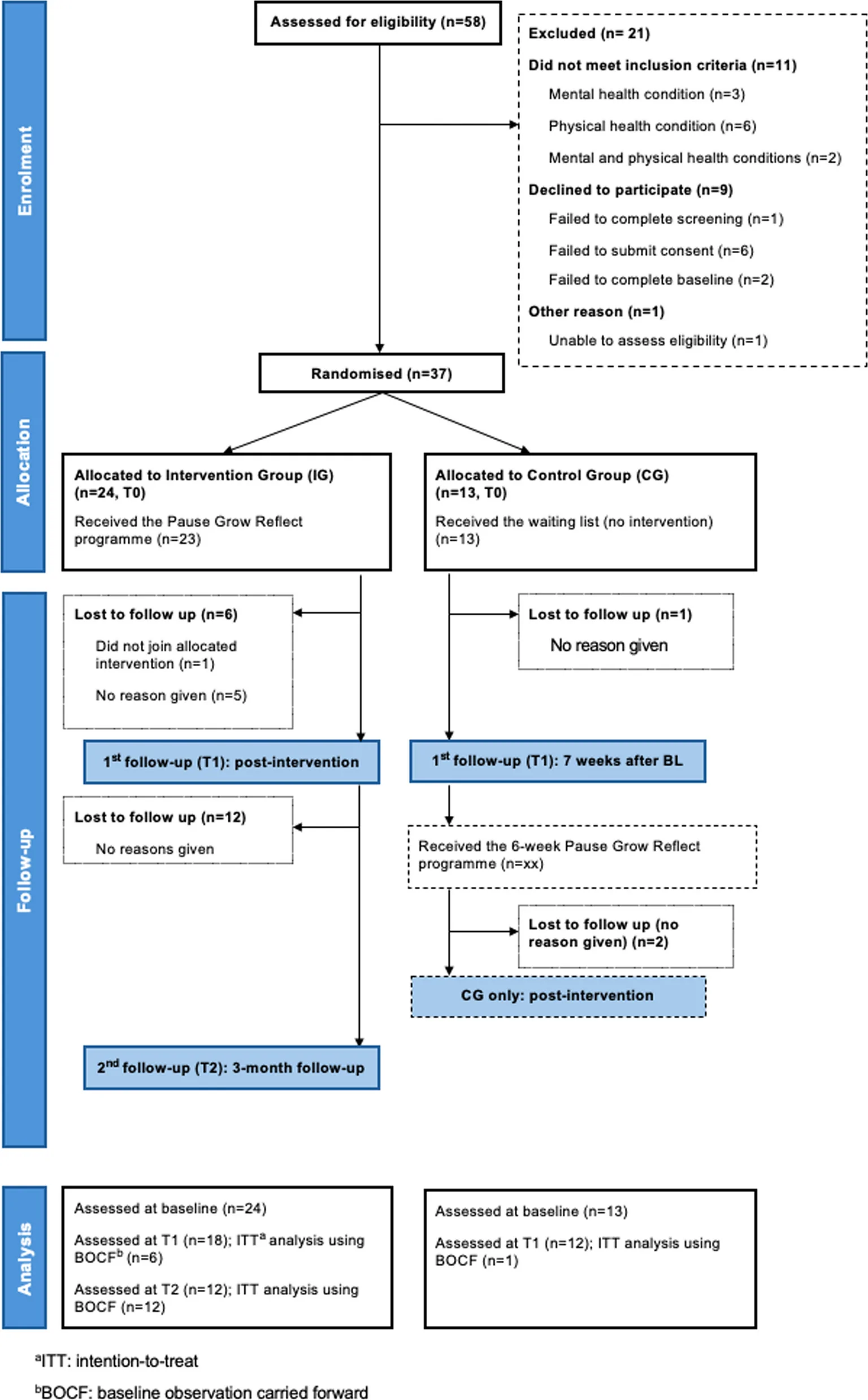
Participant flow diagram, aligned to CONSORT guidelines [41]

Thirteen of the 58 individuals who were screened were not eligible, resulting in an eligibility rate of 78% (45/58) (Fig. 2). A recruitment rate of 82% was recorded after 37 (of 45) individuals provided consent and completed T0 measures. Participants were allocated to an intervention (*n* = 24) or control (*n* = 13) group.

### Participant characteristics

Across both groups, participants were predominantly white females who identified as heterosexual and reported living without a disability, impairment, illness, or health condition, and without caring responsibilities (Table 2). Intervention group participants were aged between 23 and 60 years (M = 34.3), whilst control participants were aged 23–57 years (M = 38.8). Ten UK universities were represented in the recruited participant sample.

**Table 2:** Descriptive statistics for sociodemographic and academic characteristics

Characteristic	All participants (*n* = 37) Mean (SD) or *n* (%)	IG^a^ (*n* = 24) Mean (SD) or *n* (%)	CG^b^ (*n* = 13) Mean (SD) or *n* (%)
Age (years)	35.9 (9.83)	34.3 (8.88)	38.8 (11.2)
Gender			
Female	31 (83.8%)	19 (79.2%)	12 (92.3%)
Male	5 (13.5%)	4 (16.7%)	1 (7.7%)
Transgender, non-binary, or intersex	1 (2.7%)	1 (4.2%)	0
Sexual orientation			
Heterosexual/straight	29 (78.4%)	20 (83.3%)	9 (69.2%)
Gay or lesbian	1 (2.7%)	0	1 (7.7%)
Bisexual	3 (8.1%)	2 (8.3%)	1 (7.7%)
Other	1 (2.7%)	1 (4.2%)	0
Prefer not to say	3 (8.1%)	1 (4.2%)	2 (15.4%)
Marital status			
Single	12 (32.4%)	10 (41.7%)	2 (15.4%)
In a relationship or partnered	13 (35.1%)	8 (33.3%)	5 (38.5%)
Married or civil partnership	11 (29.7%)	6 (25.0%)	5 (38.5%)
Prefer not to say	1 (2.7%)	0	1 (7.7%)
Ethnic background			
White British or white other	27 (73.0%)	17 (70.8%)	10 (76.9%)
Asian or Asian British	3 (8.1%)	3 (12.5%)	0
Black, African, Caribbean, or black British	0	0	0
Mixed or multiple	4 (10.8%)	2 (8.3%)	2 (15.4%)
Prefer not to say	3 (8.1%)	2 (8.3%)	1 (7.7%)
Religion, faith, or belief			
Christian	9 (24.3%)	7 (29.2%)	2 (15.4%)
Buddhist	0	0	0
Hindu	1 (2.7%)	1 (4.2%)	0
Jewish	0	0	0
Muslim	2 (5.4%)	2 (8.3%)	0
Sikh	0	0	0
Spiritual but not religious	7 (18.9%)	4 (16.7%)	3 (23.1%)
Atheist	11 (29.7%)	6 (25.0%)	5 (38.5%)
Agnostic	5 (13.5%)	2 (8.3%)	3 (23.1%)
Prefer not to say	2 (5.4%)	2 (8.3%)	0
Caring responsibilities			
None	31 (83.8%)	20 (83.3%)	11 (84.6%)
Parent of child/children below age of 18	4 (10.8%)	4 (16.7%)	0
Caring for someone	1 (2.7%)	0	1 (7.7%)
Prefer not to say	1 (2.7%)	0	1 (7.7%)
Employment status			
Full-time	4 (10.8%)	4 (16.7%)	0
Part-time	13 (35.1%)	9 (37.5%)	4 (30.8%)
Zero hours or casual	5 (13.5%)	2 (8.3%)	3 (23.1%)
None	12 (32.4%)	9 (37.5%)	3 (23.1%)
Other	3 (8.1%)	0	3 (23.1%)
Highest parental qualification			
Degree level	19 (51.4%)	14 (58.3%)	5 (38.5%)
Qualifications below degree	15 (40.5%)	9 (37.5%)	6 (46.2%)
No formal qualifications	3 (8.1%)	1 (4.2%)	2 (15.4%)
Disability, impairment, illness, or health condition			
Yes	6 (16.2%)	5 (20.8%)	1 (7.7%)
No	31 (83.8%)	19 (79.2%)	12 (92.3%)
Fee status			
UK citizen	29 (78.4%)	16 (66.7%)	13 (100%)
International (within the EU)	0	0	0
International (outside the EU)	8 (21.6%)	8 (33.3%)	0
Mode of study			
Full-time	29 (78.4%)	20 (83.3%)	9 (69.2%)
Part-time	8 (21.6%)	4 (16.7%)	4 (30.8%)
Year of study			
First	11 (29.7%)	8 (33.3%)	3 (23.1%)
Second	9 (24.3%)	5 (20.8%)	4 (30.8%)
Third	11 (29.7%)	9 (37.5%)	2 (15.4%)
Fourth	3 (8.1%)	1 (4.2%)	2 (15.4%)
Fifth or above	3 (8.1%)	1 (4.2%)	2 (15.4%)
Discipline area			
Health and life sciences	19 (51.4%)	13 (54.2%)	6 (46.2%)
Engineering, environment, and computing	8 (21.6%)	4 (16.7%)	4 (30.8%)
Arts and humanities	10 (27.0%)	7 (29.2%)	3 (23.1%)
Has previous experience of yoga or yoga-related activities			
Yes	34 (91.9%)	22 (91.7%)	12 (92.3%)
No	3 (8.1%)	2 (8.3%)	1 (7.7%)
Regular yoga practice (last 3 months)^c^			
Yes	10 (29.4%)	6 (27.3%)	4 (33.3%)
No	24 (70.6%)	16 (72.7%)	8 (66.7%)
Frequency and duration of yoga practice (average)^d^			
Days per week	2.80 (1.55)	2.67 (1.97) (range 1–5)	3 (0.82) (range 2–4)
Minutes per session	40.50 (20.88) (range 20–75)	50.8 (21.1) (range 20–75)	25 (5.77) (range 20–30)
Current yoga practice or yoga-related activities^c^			
Yes	12 (35.3%)	6 (50.3%)	6 (50%)
No	22 (64.7%)	16 (72.7%)	6 (50%)

^a^*IG*, intervention group

^b^*CG*, control group

^c^Intervention group (*n* = 22); control group (*n* = 12)

^d^Intervention group (*n* = 6); control group (*n* = 4)

### Retention and follow-up rates

The retention rate across the whole sample was 32.4% (12/37), with 10 (41.7%) intervention group participants attending all six sessions. Retention decreased as the intervention progressed (Table 3). Non-attendance reasons included deadlines, childcare during holidays, vacations, personal illness, and bereavement.

**Table 3:** Participant attendance of the programme for the whole group and by trial arm, reported as *N* (%)

Number of sessions attended^a^	All (*n* = 37)	Intervention group (*n* = 24)	Control group (*n* = 13)
0	6 (16.2%)	2 (8.3%)	4 (30.8%)
1	7 (18.9%)	4 (16.7%)	3 (23.1%)
2	5 (13.5%)	4 (16.7%)	1 (7.7%)
3	3 (8.1%)	3 (12.5%)	0 (0)
4	0 (0)	0 (0)	0 (0)
5	4 (10.8%)	1 (4.2%)	3 (23.1%)
6	12 (32.4%)	10 (41.7%)	2 (15.4%)

^a^Based on session completion rate of 50% or more

Follow-up rate across the whole sample was 81% at T1. In the intervention group, one-quarter of participants (25%) did not complete T1, and half (50%) did not complete T2. In the control group, one (7.7%) and two (15.4%) participants did not complete T1 and post-intervention, respectively.

### Adherence and engagement rates

Mean percentage of pages accessed decreased as the programme progressed and ranged from 87% (7.83/9) in session one to 46% (4.58/10) in session six for the intervention group (Table 4). In the control group, access ranged from 64% (5.77/9) in session one to 19% (1.92/10) in session six.

**Table 4:** Summary of participant engagement with the Pause Grow Reflect programme overall and by group

Engagement measure	All (*n* = 37) Pages (%)	All (*n* = 37) Mean (SD)	IG^a^ (*n* = 24) Pages (%)	IG (*n* = 24) Mean (SD)	CG^b^ (*n* = 13) Pages (%)	CG (*n* = 13) Mean (SD)
Pages viewed in session 1 (range 0–9)	79.0%	7.11 (3.41)	87.0%	7.83 (2.76)	64.1%	5.77 (4.17)
Pages viewed in session 2 (range 0–10)	64.6%	6.46 (4.65)	73.8%	7.38 (4.25)	47.7%	4.77 (5.05)
Pages viewed in session 3 (range 0–9)	54.3%	4.89 (4.37)	60.2%	5.42 (4.36)	43.6%	3.92 (4.39)
Pages viewed in session 4 (range 0–9)	44.1%	3.97 (4.43)	47.2%	4.25 (4.43)	38.4%	3.46 (4.56)
Pages viewed in session 5 (range 0–9)	42.7%	3.84 (4.42)	44.9%	4.04 (4.43)	38.4%	3.46 (4.56)
Pages viewed in session 6 (range 0–10)	36.5%	3.65 (4.75)	45.8%	4.58 (5.09)	19.2%	1.92 (3.62)
Goals set across whole programme (range 0–8)	N/A^c^	2.11 (2.16)	N/A	2.42 (2.17)	N/A	1.54 (2.11)
Goal feedback given across whole programme (range 0–5)	N/A	1.43 (1.72)	N/A	1.67 (1.71)	N/A	1.00 (1.73)
Gratitude entries across whole programme^d^ (range 0–12)	N/A	1.03 (2.18)	N/A	0.79 (1.32)	N/A	0.54 (1.00)
Likes given across whole programme (range 0–10)	N/A	0.95 (1.87)	N/A	1.00 (2.15)	N/A	1.46 (3.26)
Comments posted across whole programme (range 0–11)	N/A	1.62 (2.81)	N/A	1.00 (1.14)	N/A	0.85 (1.28)
Notes created across whole programme (range 0–13)	N/A	2.92 (3.90)	N/A	4.21 (4.28)	N/A	2.77 (4.36)

^a^*IG*, intervention group

^b^*CG*, control group

^c^*N/A*, not applicable

^d^Gratitude entries available on the programme between weeks 3 and 6

### Secondary outcomes

#### Outcome measures

A marginal mean difference on scores of stress and very minimal mean differences on measures of mindful self-care, mental well-being, self-compassion, emotion regulation, mindful awareness, and resilience were observed (Table 5).

**Table 5:** Mean scores and mean difference in scores for mindful self-care, mental well-being, perceived stress, self-compassion, emotion regulation, mindful awareness, and resilience for the two groups

	Baseline (T0), mean (SD)		Post-intervention (T1), mean (SD)		Post-intervention change (T1 − T0), mean difference (95% CI)	
	IG (*n* = 24)	CG (*n* = 13)	IG (*n* = 24)	CG (*n* = 13)	IG	CG
Mindful self-care (B-MSCS)						
Mindful relaxation	2.57 (0.67)	2.27 (0.62)	2.79 (0.57)	2.50 (0.50)	0.22 (−0.09, 0.52)	0.23 (−0.12, 0.58)
Physical care	2.59 (0.70)	2.65 (0.67)	2.82 (0.66)	2.71 (0.44)	0.23 (−0.11, 0.56)	0.06 (−0.23, 0.36)
Self-compassion and purpose	2.38 (0.79)	2.29 (0.59)	2.98 (0.89)	2.37 (0.63)	0.6 (0.30, 0.91)	0.08 (−0.35, 0.51)
Supportive relationships	3.55 (0.75)	3.81 (0.75)	3.74 (0.80)	3.81 (0.67)	0.19 (−0.03, 0.41)	0 (−0.35, 0.35)
Supportive structure	3.21 (0.78)	3.62 (0.61)	3.30 (0.79)	3.71 (0.71)	0.09 (−0.27, 0.46)	0.09 (−0.26, 0.45)
Mindful awareness	2.92 (1.16)	3.26 (0.95)	3.14 (0.80)	3.23 (0.83)	0.22 (−0.15, 0.59)	−0.03 (−0.35, 0.30)
Mental well-being (SWEMWBS)	23.17 (3.81)	23.31 (4.31)	23.54 (3.96)	23.85 (3.74)	0.37 (−0.86, 1.61)	0.54 (−0.85, 1.93)
Perceived stress (PSS-10)	19.50 (5.83)	16.85 (5.89)	18.25 (6.77)	18.00 (7.34)	−1.25 (−3.12, 0.62)	1.15 (−1.38, 3.69)
Self-compassion (SCS-SF)	3.04 (0.73)	2.94 (0.86)	3.19 (0.72)	2.96 (0.98)	0.15 (0, 0.32)	0.02 (−0.18, 0.22)
Self-kindness	3.08 (0.82)	2.69 (0.90)	3.08 (0.79)	2.85 (0.99)	0 (−0.25, 0.25)	0.15 (−0.18, 0.49)
Self-judgement	3.02 (0.97)	2.81 (1.11)	3.46 (0.90)	2.65 (1.21)	0.44^a^ (0.11, 0.76)	−0.15^a^ (−0.44, 0.13)
Common humanity	3.15 (0.81)	3.12 (1.14)	3.23 (0.75)	3.08 (1.44)	0.08 (−0.19, 0.35)	−0.04 (−0.65, 0.57)
Isolation	2.94 (1.05)	3.04 (1.13)	3.00 (1.08)	2.77 (1.11)	0.06^a^ (−0.22, 0.34)	−0.27^a^ (−0.69, 0.15)
Mindfulness	3.23 (0.87)	3.19 (0.83)	3.50 (0.79)	3.81 (0.81)	0.27 (0.05, 0.50)	0.62 (0.17, 1.06)
Over-identification	2.81 (1.16)	2.77 (1.44)	2.90 (1.16)	2.58 (1.32)	0.09^a^ (−0.21, 0.37)	−0.19^a^ (−0.59, 0.21)
Emotion regulation (ERQ)						
Reappraisal	4.30 (1.37)	4.59 (1.17)	4.83 (1.11)	4.56 (1.28)	0.53 (0.20, 0.85)	−0.03 (−0.36, 0.31)
Suppression	3.65 (1.35)	3.62 (0.81)	3.66 (1.28)	3.65 (0.95)	0.01 (−0.33, 0.35)	0.04 (−0.42, 0.50)
Mindful awareness (MAAS)	3.37 (1.07)	3.76 (0.92)	3.44 (0.99)	3.83 (0.78)	0.07 (−0.16, 0.31)	0.07 (−0.32, 0.45)
Resilience (BRS)	3.21 (0.79)	3.33 (0.79)	3.35 (0.72)	3.09 (1.00)	0.14 (−0.04, 0.32)	−0.24 (−0.50, 0.01)

^a^Reversed scoring meaning that a higher mean difference was better

### Adverse events

Six negative unintended effects were reported during or directly after yoga sessions across the whole group and related to instruction moving too quickly, difficulty with nasal breathing, feelings of frustration or inadequacy whilst trying to follow the video, and concerns about pose difficulty.

### Intervention acceptability: quantitative

Across the whole group, most (83%) participants viewed the intervention as acceptable, with the majority of participants (77%) reporting liking the programme and almost two-thirds (63%) of participants reporting it had improved their self-care behaviour. Around three-quarters (77%) of participants felt it was clear how the programme intended to improve self-care behaviour, more than half (57%) of participants felt confident about engaging in the programme, and most (53%) disagreed that there were moral or ethical consequences to engaging. Half of participants (53%) felt it demanded ‘a little effort’, whilst four-in-ten (40%) reported that engaging in Pause Grow Reflect interfered with other priorities. Full responses are presented in Supplementary Material 5.

### Intervention acceptability: qualitative

Ten participants took part in an interview, lasting 36–64 min (M = 48 min). Seven (70%) interviewees were female, with ages ranging from 25 to 49 years (M = 36.4 years). Half of the participants identified as white or white British, with the remaining participants from Asian and mixed or multiple ethnic backgrounds. Participants were UK citizens or international students from outside the European Union (50% each). Most participants reported no caring responsibilities (70%), were not employed (80%), and did not report a disability, illness, or health condition (80%). All seven TFA constructs were identified, with sub-themes and illustrative quotes (Table 6).

**Table 6:** An outline of themes and sub-themes generated

TFA domain	Sub-themes
General acceptability	
Affective attitude	• Pause Grow Reflect enabled the learning new skills with tools to support enactment • Navigating the pros and cons of a digital intervention • Support and guidance to support enjoyment and engagement
Burden	• Capacity and effort required to participate in the intervention • Issues with remembering to complete the intervention • Methods of improving the intervention for PGRs
Ethicality	• Connection to the content and activities • Ethicality of the spiritual content • Cultivating greater connection to the intervention
Perceived effectiveness	• Encouraged self-reflection and self-awareness • Promoted change in day-to-day practices • Physical, mental, and social benefits • Beyond Pause Grow Reflect
Intervention coherence	• Perceptions of the purpose of Pause Grow Reflect • Perceived purpose and scope of intervention components
Opportunity costs	• Difficulties prioritising the programme • Ease of implementing certain activities
Self-efficacy	• Self-efficacy in completing Pause Grow Reflect • Confidence in completing the practical yoga sessions • Confidence in performing the reflection tools and practices

#### General acceptability

Many participants expressed how ‘worthwhile’ the intervention was for them and considered the intervention to be of high value to them, indicating overall acceptability.

*It’s amazing. This programme is really, really amazing and really helpful, so I love this programme. I hope I can join another programme like this.* (Participant 1)

#### Affective attitude

Affective attitude refers to participants’ attitudes and feelings towards participating in the programme.

##### Pause grow reflect enabled learning new skills with tools to support enactment

Participants described the weekly yoga sessions as enjoyable, helping them to ‘de-stress’ and ‘relax’. The intervention allowed some participants the opportunity to try yoga or a different yoga style for the first time, and useful activities and tools. The order and format of the sessions encouraged a sense of growth and inspiration to learn about yoga’s history, philosophy, and underpinning evidence base.

*coming back every week and being taught something new... it felt like growth because every week it built upon the previous week* (Participant 7)

##### Navigating the pros and cons of a digital intervention

The digital form of delivery offered greater flexibility and accessibility, and an opportunity to practise in an environment of their choosing. It was visually displayed in an acceptable way (e.g. layout, bookmark feature, gamification). However, participants often struggled to find a suitable, relaxing space to practise and were unable to receive real-time feedback given the asynchronous delivery.

*I always think that having like the little like medals and achievements kind of that gamification is really, really cool because you kind of go, great I’ve done my little um goal for the week* (Participant 10)

*And I know when you have a physical space... it’s all about the ambience as well... And that’s just not something that we can feasibly control* (Participant 8)

##### Support and guidance prompting enjoyment and engagement

Participants valued the tone and language used, allowing participants to exercise agency over the extent of their participation, and highlighted how the qualities of non-judgement, compassion, care, and understanding improved their enjoyment and engagement with the intervention.

*you’re practising your philosophy and how you’re talking to us throughout the course in that you’re allowing people not to feel that they have to do everything* (Participant 5)

#### Burden

Burden refers to the amount of perceived effort that was required to participate.

##### Capacity and effort required to participate in the intervention

Participants reported the amount of effort and time required to engage in the educational and reflective components as burdensome. Although considered worthwhile, the self-led nature of the intervention meant participants had to prioritise and manage their time, which felt like another responsibility.

*some aspects of it, asking you to do more thinking and writing, for me that feels like, oh I can’t do that, I’m sorry. That was part of my PGR experience, was that my brain felt overloaded, and it couldn’t take on more* (Participant 5)

##### Issues with remembering to complete the intervention

Participants sometimes forgot to complete intervention activities (notably the practical yoga sessions and social community space). A limited number of prompts, notifications received at inconvenient times (e.g. during working hours) or through unhelpful methods (e.g. via email), and issues with scheduling activities were reported. Acknowledging difficulties in remembering to engage, several participants suggested receiving notifications through platforms like WhatsApp or calendar invites.

*giving them an option to set a reminder for doing that session… I think having that kind of integration into maybe a calendar or something would be very useful* (Participant 10)

##### Methods of improving the intervention for postgraduate researchers

Suggestions included offering real-time sessions with the facilitator or shorter sessions to promote engagement and bring participants together. Participants also recommended more engaging formats (e.g. audio or video), making some content optional, and better integration of the social community.

*Like it [reading the educational content] felt like such a drag and like I would have much rather I had one video to watch* (Participant 4)

#### Ethicality

Ethicality reflects the extent to which the Pause Grow Reflect programme has a good fit with participants’ value systems.

##### Connection to the content and activities

Most participants expressed that they felt connected to, and valued, the content and materials. Some participants described that the content aligned with how they already had intentions to incorporate into their day-to-day lives or were already doing (e.g. approaching others/oneself with kindness).

*in terms of the programme it’s pulled together the feelings and the thoughts that I was having* (Participant 7)

##### Ethicality of the spiritual content

Some participants expressed intentions to find strategies to complement their religious beliefs and faith. Despite some participants showing less interest in this content, participants expressed that they were still open to learning. Others described how the intervention might address misconceptions and preconceived notions, with one participant suggesting testimonials emphasising the benefits of mind-body practices could encourage uptake.

*because it [Pause Grow Reflect] also touched [on] the spiritual aspects… I just want to like broaden my vision and not just stick to only my values and only my beliefs* (Participant 6)

*if you’ve done it and that’s really helping you, it could maybe help me* (Participant 10)

##### Cultivating greater connection to the intervention

For participants to feel more connected and motivated, some reported that meeting the facilitator at the beginning and adjusting the intervention to be more tailored to the specific experiences and challenges postgraduate researchers encounter (e.g. imposter syndrome, procrastination) could be beneficial.

*some of the things that are common for PhD students like imposter syndrome and linking in with, you know, procrastination… maybe could have sparked some more community conversation* (Participant 9)

*if I had met you [facilitator] from the beginning that I may have been more inclined to try a bit more* (Participant 4)

#### Perceived effectiveness

Perceived effectiveness reflects the extent to which participants perceive the Pause Grow Reflect intervention as likely to achieve its purpose.

##### Encouraged self-reflection and self-awareness

Many participants expressed self-reflection, prompting an awareness of what they were already doing and what to change or implement in their lives. The knowledge and experience of self-care and yoga enabled participants to reflect on tools and strategies they could use.

*it’s always a reminder for me that… there’s lots of things, and there’s lots of strategies that you that you can do to cope with these stress[ors]* (Participant 6)

##### Promoted change in day-to-day practices

Pause Grow Reflect reportedly prompted participants to implement self-care through breathing techniques to approach situations with more compassion and understanding. Some participants also reported taking breaks and developing healthier working patterns. The goal setting, feedback, and self-reward support were helpful. In contrast, one participant expressed difficulty adopting strategies, despite now knowing what they needed to address.

*I’ve been getting up quite a bit earlier but not just going into stressful work straight away and taking a few moments doing some breathing practice, doing something more relaxing and then starting work* (Participant 3)

##### Physical, mental, and social benefits

Participants reported experiencing improvements to their sense of connection and mental and physical well-being, including sleeping patterns, mindful awareness, improved breathing practice, flexibility, stress management, and feeling more relaxed or refreshed. One participant explicitly reported that the amount of intervention content and effort prevented benefits.

*It’s bring[s] a lot of benefits for me, especially for myself, about my movement, about my flexibility, about my, how to reduce my stress and then how to handle my emotional issue* (Participant 1)

##### Beyond pause grow reflect

Many participants expressed their intention to continue to practise yoga and/or self-care beyond Pause Grow Reflect. This included engaging in a physical yoga practice as well as continuing practices such as self-reflection, mindfulness, and learning more.

*it allowed me to go that’s why I think that way and that’s why I’m doing that… I intend to carry on in some way afterwards as well* (Participant 7)

#### Intervention coherence

Intervention coherence reflects the extent to which participants understood the Pause Grow Reflect intervention and how it worked.

##### Perceptions of the purpose of pause grow reflect

Many participants understood the intervention intended to help postgraduate researchers to manage stress, support and enhance their well-being, and/or help participants to implement self-care behaviours. There was recognition that the intervention encouraged participants to explore different aspects of self-care (e.g. mental, emotional, spiritual) and yoga (e.g. yoga philosophy and yogic principles).

*I’m really glad that it wasn’t just about the movement. I’m glad the background information was there as well* (Participant 3)

##### Perceived purpose and scope of intervention components

Some participants expressed a lack of clarity around the scope and capacity of the facilitator’s role. Uncertainty around the purpose and importance of engaging in activities (e.g. weekly goal setting and reflection) led some participants to engage less.

*we can contact you directly if we feel like overwhelmed. Umm I think that but I was like very, not sure that I can like use it* (Participant 6)

#### Opportunity costs

Opportunity costs refer to the extent to which benefits, profits, or values had to be given up in order to engage in the Pause Grow Reflect intervention.

##### Difficulties prioritising pause grow reflect

Engaging in the programme influenced other daily activities and was at times challenging to complete. This was apparent for participants who had family commitments and competing demands (e.g. employment) alongside their PhD. Prioritisation tended to fluctuate based on personal circumstances and demanding periods.

*in actual reality the priority is the writing [for the PhD], and creating the space and time to do the writing because I need to get it done. And then the kids, all of that stuff comes to a head* (Participant 5)

##### Ease of implementing certain activities

Participants shared experiences of how they completed intervention components alongside other activities (e.g. whilst travelling or watching TV), suggesting that certain activities may be easier to implement and less likely to interfere with daily activities.

*sometimes that I opened er the platform… in my headphones when I’m on the bus* (Participant 1)

#### Self-efficacy

Self-efficacy refers to participants’ ability to complete the Pause Grow Reflect intervention and perform the behaviours required to participate in the programme.

##### Self-efficacy in completing pause grow reflect

Many participants reported feeling able to complete the sessions, finding most aspects of the intervention easy to use, navigate, and understand. Although participants’ confidence fluctuated based on expectations and during busier weeks.

*I’ve actually started off more confident and towards the middle I thought oh, maybe I’m not as agile and flexible and able to switch things off as much as I thought* (Participant 7)

##### Confidence in completing the practical yoga sessions

Several participants felt initially less confident about the physical yoga postures and practices (e.g. alternate nostril breathing). Concerns pertained to doing the postures ‘wrong’, expectations, difficulties with nasal breathing, and a lack of prior experience. Despite this, the guidance provided was reassuring and necessary. Recommendations included optional live yoga sessions to receive real-time feedback and verbal adjustments, or a resource with diagrams to encourage familiarisation.

*I think there was one [breathing practice] where we had to breathe through one side of the nose and not the other and ohh that one was very difficult* (Participant 9)

*if I had had maybe some diagrams or some like brief explanation of each individual pose so, to sort of know a bit more before going into it* (Participant 3)

##### Confidence in performing the reflection tools and practices

Several participants revealed that they were more confident reflecting on ideas and topics they were already familiar with, whilst other participants reported feeling confused due to the length and complexity. One participant suggested breaking the questions down into shorter, simpler questions.

*more challenging… it wasn’t something I was conscious of, and it wasn’t something I’ve thought about* (Participant 9)

## Discussion

### Summary of findings

This trial assessed the feasibility and acceptability of Pause Grow Reflect, a multicomponent yoga-based intervention targeting improved self-care and mental well-being in postgraduate researchers. Findings showed measures of recruitment and follow-up at T1 met the progression criteria, whilst follow-up at T2 and rates of retention and engagement did not. Minimal harm was reported with responses pertaining to difficulties following yoga instruction, challenging thoughts or feelings, and the need for further modifications and options. The qualitative findings indicated a favourable evaluation across most of the TFA constructs, revealing alignment with the quantitative data. Pause Grow Reflect was a unique and valued programme for participants with almost three-quarters of participants reporting that they liked the intervention.

### Strengths and limitations

Given the paucity of online interventions specifically developed to support mental well-being and self-care in postgraduate researchers during their studies, a strength of this study was the use of mixed methods and the application of the TFA to gain a better understanding of intervention acceptability of an intervention designed specifically for postgraduate researchers. The inclusion of a waitlist control group allowed for comparative analysis of recruitment, retention, follow-up, adherence, and engagement rates. Another strength was that half of the postgraduate researchers interviewed were international students from outside the EU, representing a group often underrepresented in research in this area. The main limitation of this study was the homogeneous sample (based on gender, ethnic background), which limits the generalisability of findings to male and non-white postgraduate researchers, which is commonly reported in research on self-care and/or yoga. As this study was not powered, as is typical in feasibility studies, only limited conclusions can be drawn from the positive preliminary efficacy findings. The interview data were collected from a self-selecting, convenience sample. Therefore, we may have mainly captured the views of those interested in the intervention, with only one participant attending less than half of intervention sessions. A final potential limitation was that a few participants were familiar to the main researcher (NB) which could have influenced responses and resulted in more favourable results. However, constructive comments to support intervention refinement were provided suggesting the rapport was beneficial to allow open and honest feedback.

### Interpretation of findings and consistency with the literature

Rates of retention, adherence, and declining engagement did not meet the progression criteria is in line with previous research alluding to the challenges of retaining and engaging participants [58]. Reviews on digital mind-body interventions (including yoga and mindfulness) have demonstrated that adherence, engagement, and retention can represent barriers to such interventions [59]. Similar patterns have been demonstrated in reviews of mindfulness interventions with reports of relatively high attrition and drop-out rates [60]. Potential reasons for the declines (e.g. lack of time) correspond with barriers identified in previous studies exploring online or app-based interventions with university students [61, 62], as well as various survey studies exploring barriers to self-care and yoga participation [26, 63]. Survey findings have demonstrated that a lack of time, motivation, and energy are commonly reported barriers to engaging in self-care strategies among graduate psychology students [26] and similarly feeling busy, pressured for time, and overscheduled acted as barriers to yoga participation in university students [63]. These findings are unsurprising considering the already demanding nature of doctoral-level study. The timepoint at which an intervention of this kind is offered is therefore an important consideration.

Although there have been mixed findings around facilitated versus self-led digital psychological and mind-body interventions for stress management and/or mental well-being [30, 64–66], this study demonstrated that the facilitator support along with the tone and approach of the intervention overall was important. Participants also liked the digital platform (including features, look-and-feel) and found it convenient, accessible, and flexible, which was likely to be imperative to accommodate demanding workloads, busy schedules, limited time, and financial constraints. These findings are consistent with other findings which demonstrate that digital interventions can be more accessible, less costly in terms of travel, and highly scalable [30]. Several studies have also found that face-to-face and online mind-body interventions are similar in terms of acceptability and effectiveness with online delivery potentially more effective for maintenance of health and well-being [67, 68]. Online delivery also enables participants to engage with specific components that interest them [45]. This aligns with a yoga study conducted with medical students recommending the availability of tailored and customisable options [62]. The potential value of digital interventions to support postgraduate researcher self-care and well-being during their studies is thus demonstrated.

Limitations to asynchronous delivery included the absence of real-time guidance and feedback from the yoga teacher, lack of accountability, effort required to participate in specific aspects of the programme, no sense of social community, and external constraints. Similar findings were found in other online yoga interventions across a wide range of study populations where participants reported being unable to receive guidance on adjustments that would otherwise be available in in-person classes [68–70], a lack of social community, support and/or connection [68, 70, 71], and physical constraints such as distractions and/or finding a quiet place to practise mind-body tools [68, 71]. Findings from an online yoga intervention delivering synchronous yoga revealed good attendance and positive experiences, likely benefiting from the range of classes offered (three morning and three evening classes per week) [70]. It is evident that doctoral-level study is highly independent and could place heavy demands on students’ psychological resources. Together with difficulties of decision fatigue, procrastination, and limited mental resources, identified by participants in the current study, predominantly self-led online programmes may not always be favoured.

Social support is an important factor for postgraduate researcher well-being with loneliness and isolation reported in doctoral-level study [72]. Social support can increase enjoyment, adherence, and attendance in behavioural interventions [73]. In the current study, participants suggested various reasons a sense of community and connection was lacking, and these were consistent with a previous study which assessed mentoring, project planning, and mindfulness in enhancing postgraduate researcher mental health and well-being [61]. Low engagement with an online peer forum in that study was attributed to limited platform functionality but facilitator-led workshops did prompt social interaction [61]. In a RCT conducted with university students assessing an online multicomponent mindfulness intervention, findings revealed no participation in the asynchronous discussion forums [74]. The researchers suggested greater interest in self-learning and a potential redundancy due to other messaging platforms [74]. Although the co-creation phase of this research (described previously) likely increased intervention acceptability, more research must address the barriers to accessing peer support and to understand which method of delivery and platforms would promote better adherence and engagement within postgraduate researchers.

## Conclusion

This study explored the feasibility and acceptability of incorporating self-care and yoga to promote postgraduate researcher mental well-being and self-care behaviours. Findings demonstrated Pause Grow Reflect, a multicomponent digital intervention, was feasible to deliver and evaluate among UK-based students, and mostly acceptable. Further refinements are needed prior to a definitive trial, focusing on improvements to address retention, adherence, and engagement. Refinement efforts should ensure more diverse input to ensure these further refinements are reflective of the wider postgraduate researcher population.

## Supplementary Information

Additional file 1. Supplementary materials 1 to 5.

Additional file 2. CONSORT 2010 checklist.

## Data Availability

The data generated and analysed for this study are not publicly available as consent was not provided by participants to make their anonymised data publicly available alongside the manuscript.
