# Supplementary material for: A digital yoga-based ‘Pause Grow Reflect’ intervention for postgraduate researchers: a feasibility randomised controlled trial and embedded qualitative acceptability study
Source: Pilot Feasibility Stud. 2026 Jun 12;12:122. doi: 10.1186/s40814-026-01807-9 (PMC13495291; doi:10.1186/s40814-026-01807-9)
Supplement: Supplementary file 2 [file 40814_2026_1807_MOESM1_ESM.docx]

**Supplementary Material 1**.

**An overview of the intervention sessions and the topics covered.**

| **Session** | **Aim** | **Main content/topics** | **Activities** |
| --- | --- | --- | --- |
| Pre-session:  Pause Grow Reflect Introduction | - To introduce the programme and H4C platform - To introduce oneself to the cohort | - Introduction to content, structure, expectations - Using the platform - Facilitator and support - Community guidelines and safety | - *Social community/Forum*: ‘introducing yourself’, hopes/intentions/motivations for programme - Set up profile |
| Session 1: Understanding self-care and yoga | - To develop an awareness of self-care, well-being, and yoga in a PGR context with a focus on grounding techniques to support PGRs' well-being | - Introduction to self-care, well-being, yoga (incl. the 8-Limbs of Yoga) - Introduction to self-awareness, self-reflection, and self-regulation - Interconnections between kosha model and BPSS^b^ model - Self-compassion and Ahimsa (yogic principle meaning ‘non-harm’) - Overview of SMARTER goal approach | - Psychoeducation, educational video - SMARTER^a^ goal setting - *Self-inquiry and reflection*: self-compassion and Ahimsa in own life - *Yoga session 1*: grounding and rooting (theme), extended exhale breathing practice, visualisation guided meditation - *Social community/Forum*: how did you find week one’s grounding practice and/or reflections? Do you have reflections or take aways to share? |
| Session 2: The Physical Layer of experience | - To develop an awareness of the physical body and physical self-care needs and to understand stress and the stress response | - Introduction to physical kosha and self-care behaviours (e.g. basic human needs: sleep, diet/nutrition, movement, rest, posture) - Understanding stress, different types of stress, and the stress response - Introduction to physical yoga practice and benefits - Introduction to Satya (yogic principle meaning ‘truthfulness’) | - Psychoeducation, educational video - SMARTER^a^ goal setting - Goal feedback (from week one) - *Self-inquiry and reflection*: identifying basic physical needs and incorporating into self-care practice; Satya in own life - *Yoga session 2:* awareness and observation of body and physical sensations (theme), three-part (Dirga) breathing practice, body scan - *Social community/Forum*: how did you find week two’s practice and/or reflections? Do you have reflections or take aways to share? |
| Session 3: The Energetic Layer of experience | - To develop an awareness of the breath and breathing practices and to identify signs of stress and stressors or challenges in one's life | - Introduction to energetic kosha and the breath - Developing awareness of common signs of stress and stressors/challenges of PGRs across Self-Care Domains - Introduction to breathing practices (pranayama) in yoga and benefits - Introduction to Brahmacharya (yogic principle meaning ‘non-excess’) | - Psychoeducation, educational video - SMARTER^a^ goal setting - Goal feedback (from week two) - *Self-inquiry and reflection*: own experiences of stress and main stressors or challenges as a PGR; Brahmacharya in own life - *Yoga session 3*: awareness and observation of the breath (theme), alternate nostril (Nadi Shodhana) breathing practice, guided meditation - *Social community/Forum*: how did you find week three’s practice and/or reflections? Do you have reflections or take aways to share? |
| Session 4: The Mental and Emotional Layer of experience | - To develop an awareness of the mind (thoughts and emotions) and mindfulness practices, and to understand and identify perceptions of stress and coping strategies | - Introduction to mental and emotional kosha, aspects of the mind, self-care behaviours - Introduction to notion of awareness and thoughts, emotions, beliefs - Perceived stress and coping strategies/ styles - Introduction to mindfulness and open-monitoring practice in yoga and benefits - Introduction to Asteya (yogic principle meaning ‘non-stealing’) | - Psychoeducation, educational video - SMARTER^a^ goal setting - Goal feedback (from week three) - Gratitude post - *Self-inquiry and reflection*: own coping strategies/styles; Asteya in own life - *Yoga session 4*: awareness and observation of the mind (theme), Victorious (Ujjayi) breathing practice, intention/affirmation guided mindfulness meditation - *Social community/Forum*: how did you find week four’s practice and/or reflections? Do you have reflections or take aways to share? |
| Session 5: The Wisdom and Intellectual Layer of experience | - To develop an awareness of the intellectual or 'higher' mind (i.e., wisdom, discernment, insight, awareness) and meditation practice, and to understand self-reflection and self-awareness | - Introduction to wisdom and intellectual kosha and aspects of the observing mind - Introduction to concepts of awareness, discernment, discriminative wisdom - Introduction to values and virtues - Introduction to focused attention and concentration practice in yoga and benefits - Introduction to Svadhyaya (meaning ‘self-study’) and Aparigraha (meaning ‘non-attachment’) | - Psychoeducation, educational video - SMARTER^a^ goal setting - Goal feedback (from week four) - Gratitude post - *Self-inquiry and reflection*: own values; Aparigraha in own life - *Yoga session 5*: awareness, focus (Drishti) and concentration (theme), Bee breath (Bhramari) breathing practice, focused attention guided meditation - *Social community/Forum*: how did you find week five’s practice and/or reflections? Do you have reflections or take aways to share? |
| Session 6: The Spiritual (or Bliss) Layer of experience | - To develop an awareness of connection (to self, others, nature, something greater) and awareness or consciousness, and to explore concepts of joy and contentment | - Introduction to spiritual or bliss kosha and related self-care behaviours - Introduction to concepts of conscious awareness, connection, joy, contentment - Introduction to meditation practice in yoga and benefits   Introduction to Tapas (meaning ‘self-discipline’) and Santosha (meaning ‘contentment’) | - Psychoeducation, educational video - SMARTER^a^ goal setting - Goal feedback (from week five) - Gratitude post - *Self-inquiry and reflection*: Tapas and Santosha in own life - *Yoga session 6*: contentment, connection, and joy (theme), ‘breath of joy’ breathing practice, guided meditation |

**Supplementary Material 2.**

**A completed Template for Intervention Description and Replication (TIDieR) checklist (Hoffmann et al., 2014) for Pause Grow Reflect.**

| **Item no.** | **Item** | **Evidence** | **Location (section in paper)** |
| --- | --- | --- | --- |
| 1. | **Brief name**  Provide the name or a phrase that describes the intervention. | Pause Grow Reflect: a digital yoga-based self-care intervention to promote self-care behaviour and positive mental well-being in postgraduate researchers | Methods: Intervention group |
| 2. | **Why**  Describe any rationale, theory, or goal of the elements essential to the intervention. | Self-care through a yoga intervention tailored to PGRs may encourage self-care behaviour, better stress management and improved mental well-being through increasing PGR capability, opportunity and motivation to self-care as well as through promoting healthy coping strategies and mind-body tools and strategies for stress response. | Introduction |
| 3.  4. | **What**   - Materials: Describe any physical or informational materials used in the intervention, including those provided to participants or used in intervention delivery or in training of intervention providers. Provide information on where the materials can be accessed (e.g. Online appendix, URL) - - Procedures: Describe each of the procedures, activities, and/or processes used in the intervention, including any enabling or support activities | **Materials:** Participants received a 6-week yoga-based self-care intervention. Participants were given access to the online platform (for 7 weeks in total) where the intervention content was held.  **Procedures:** A range of yoga-based practices and psychoeducational information for PGRs formed part of the intervention. This included formal practices (with accompanying written material, audio, videos; including intention setting and grounding, breathing practice, postural practice, and relaxation and meditation), weekly activities (e.g., goal setting, gratitude), 10-15 minute reflection (accompanied by a short meditation or breathing practice) and psychoeducational information. New content was released to participants at the beginning of each week. | Methods: Participants, Intervention Group |
| 5. | **Who provided**  For each category of intervention provider (e.g. Psychologist, nursing assistant), describe their expertise, background and any specific training given. | Delivered by the main researcher (NB) who is a qualified yoga instructor with a number of years’ teaching experience and additional training in yin yoga and yoga therapy for anxiety module. She produced audio/visually recorded content and uploaded, as well as offered prompts to participate in the community forum. She received training to facilitate on the H4C platform. The wider H4C team supported in intervention setup, administration and handled technical issues. | Methods: Intervention group |
| 6. | **How**  Describe the modes of delivery (e.g. Face-to-face or by some other mechanism, such as internet or telephone) of the intervention and whether it was provided individually or in a group | The intervention was delivered online using the H4C platform to a group of participants in a cohort. | Methods: Intervention group |
| 7. | **Where**  Describe the type(s) of location(s) where the intervention occurred, including any necessary infrastructure or relevant features | Online (remotely). Participants were required to have access to the internet and to a device that allowed them to access the intervention platform (e.g., computer/laptop, tablet, or smartphone). Participants were able to use the intervention from any place them chose and needed enough space to do the activities. | Methods: Intervention group |
| 8. | **When and how much**  Describe the number of times the intervention was delivered and over what period of time including the number of sessions, their schedule, and their duration, intensity or dose. | Delivered over 6 weeks. Participants were encouraged to complete one formal yoga practice and a 10-15 minute reflection session per week as a minimum along with short weekly tasks (i.e. goal setting, gratitude post) at a time that suited them. | Methods: Intervention group |
| 9. | **Tailoring**  If the intervention was planned to be personalised, titrated or adapted, then describe what, why, when, and how. | N/A | N/A |
| 10. | **Modifications**  If the intervention was modified during the course of the study, describe the changes (what, why, when, and how). | All participants were given an additional, non-facilitated week of access to the intervention to enable participants more time to finish the intervention. | Methods: Intervention group |
| 11.  12. | **How well**   - Planned: If intervention adherence or fidelity was assessed, describe how and by whom, and if any strategies were used to maintain or improve fidelity, describe them - Actual: If intervention adherence or fidelity was assessed, describe the extent to which the intervention was delivered as planned | Planned: intervention fidelity and adherence assessed.  Actual: intervention fidelity and adherence reported on. | Methods: Secondary outcomes, Intervention fidelity |

**Supplementary Material 3.**

**Interview Topic Guide for the ‘Pause Grow Reflect’ study**

**Introduction to interview:**

**Introduce yourself, check consent, understanding of the purpose of interview, whether they are happy for the interview to be recorded, and understand right to withdraw.**

<Questions based on the Theoretical Framework of Acceptability (TFA)>

**TFA domain: Affective; how an individual feels about an intervention**

Can you tell me how you feel about the PGR study/intervention?

- What did you like most/like the least?
- How does this intervention compare to anything else you may have tried?

What are your thoughts around the digital delivery of the PGR intervention?

**TFA domain: Burden; perceived amount of effort required**

How much effort was required for you to participate in the intervention?

- How convenient was it for you to participate

How convenient was it accessing the digital platform?

- Ease of navigation
- Learning to use the app/platform

**TFA domain: Ethicality; extent to which the intervention has good fit with an individual’s value system**

How appropriate did you find the intervention content?

- Was it something you connected with/did not connect with
- Do you have any ethical concerns with the intervention

**TFA domain: Intervention Coherence; extent to which the participant understands the intervention and how it works**

Can you tell us about how you think the intervention works?

- What was the purpose of the intervention in your opinion?
- What information did you receive about the intervention?
- What were your expectations of the intervention?

**TFA domain: Opportunity costs; extent to which benefits, profits or values must be given up to engage in the intervention**

How much of a priority was it for you to engage with the intervention?

- Was it easy/difficult apply the practices/tools learned within the intervention within your day-to-day?

**TFA domain: Perceived effectiveness extent to which the intervention is perceived as likely to achieve its purpose**

How do you feel the intervention has helped/not helped you?

- What have been the benefits for you?
- What could have been improved to better meet your needs?

**TFA domain: Self-efficacy confidence that they can perform the behaviours required to participate in the intervention**

How confident do you feel about your ability to perform the behaviours and practices you have learned through the intervention?

- How confident were you in your ability to engage with the intervention?
- Did you feel like you would need more support to engage with the intervention content?

General acceptability – How would you describe your overall experience of the intervention?

Opportunity for final comments about the intervention.

**Supplementary Material 4.**

**Pre/post measures**

Table. Descriptive statistics for outcome measures at baseline (T0), post-intervention (T1), and follow-up (T2) by trial arm, reported as mean (SD).

|  | **Intervention Control**  **group (n=24) group (n=13)** | | |  |
| --- | --- | --- | --- | --- |
|  | **T0** | **T1^a^** | **T0** | **T1^b^** |
| **Mindful self-care**  Mindful relaxation  Physical care  Self-compassion and purpose  Supportive relationships  Supportive structure  Mindful awareness | 2.57 (0.67)  2.59 (0.70)  2.38 (0.79)  3.55 (0.75)  3.21 (0.78)  2.92 (1.16) | 2.79 (0.57)  2.82 (0.66)  2.98 (0.89)  3.74 (0.80)  3.30 (0.79)  3.14 (0.80) | 2.27 (0.62)  2.65 (0.67)  2.29 (0.59)  3.81 (0.75)  3.62 (0.61)  3.26 (0.95) | 2.50 (0.50)  2.71 (0.44)  2.37 (0.63)  3.81 (0.67)  3.71 (0.71)  3.23 (0.83) |
| **Mental wellbeing** | 23.17 (3.81) | 23.54 (3.96) | 23.31 (4.31) | 23.85 (3.74) |
| **Perceived stress** | 19.50 (5.83) | 18.25 (6.77) | 16.85 (5.89) | 18.00 (7.34) |
| **Self-compassion**  Self-kindness  Self-judgement  Common humanity  Isolation  Mindfulness  Over-identification | 3.04 (0.73)  3.08 (0.82)  3.02 (0.97)  3.15 (0.81)  2.94 (1.05)  3.23 (0.87)  2.81 (1.16) | 3.19 (0.72)  3.08 (0.79)  3.46 (0.90)  3.23 (0.75)  3.00 (1.08)  3.50 (0.79)  2.90 (1.16) | 2.94 (0.86)  2.69 (0.90)  2.81 (1.11)  3.12 (1.14)  3.04 (1.13)  3.19 (0.83)  2.77 (1.44) | 2.96 (0.98)  2.85 (0.99)  2.65 (1.21)  3.08 (1.44)  2.77 (1.11)  3.81 (0.81)  2.58 (1.32) |
| **Emotion regulation**  Reappraisal  Suppression | 4.30 (1.37)  3.65 (1.35) | 4.83 (1.11)  3.66 (1.28) | 4.59 (1.17)  3.62 (0.81) | 4.56 (1.28)  3.65 (0.95) |
| **Mindful awareness** | 3.37 (1.07) | 3.44 (0.99) | 3.76 (0.92) | 3.83 (0.78) |
| **Resilience** | 3.21 (0.79) | 3.35 (0.72) | 3.33 (0.79) | 3.09 (1.00) |

*Note. T0: baseline; T1: post-intervention.*

*^a^BOCF: baseline observation carried forward, n=6 additional*

*^b^BOCF: baseline observation carried forward, n=1 additional*

**Supplementary Material 5.**

**TFA questionnaire responses**

Responses to TFA question “*Did you like or dislike the Pause Grow Reflect programme?*”

| Rating | **All participants (n=30)**  n (%) | **IG (n=19)**  n (%) | **CG (n=11)**  n (%) |
| --- | --- | --- | --- |
| Strongly like | 12 (40.0%) | 9 (47.4%) | 3 (27.3%) |
| Like | 11 (36.7%) | 5 (26.3%) | 6 (54.5%) |
| No opinion | 3 (10.0%) | 2 (10.5%) | 1 (9.1%) |
| Dislike | 4 (13.3%) | 3 (15.8%) | 1 (9.1%) |
| Strongly dislike | 0 | 0 | 0 |

Responses to TFA question “*How much effort did it take to engage with the Pause Grow Reflect programme?*”

| Rating | **All participants (n=30)**  n (%) | **Intervention group (n=19)**  n (%) | **Control group (n=11)**  n (%) |
| --- | --- | --- | --- |
| No effort at all | 0 | 0 | 0 |
| A little effort | 16 (53.3%) | 10 (52.6%) | 6 (54.5%) |
| No opinion | 1 (3.3%) | 1 (5.3%) | 0 |
| A lot of effort | 11 (36.7%) | 7 (36.8%) | 4 (36.4%) |
| Huge effort | 2 (6.7%) | 1 (5.3%) | 1 (9.1%) |

Responses to TFA question “*There are moral or ethical consequences to engaging with the Pause Grow Reflect programme*”

| Rating | **All participants (n=30)**  n (%) | **Intervention group (n=19)**  n (%) | **Control group (n=11)**  n (%) |
| --- | --- | --- | --- |
| Strongly disagree | 8 (26.7%) | 5 (26.3%) | 3 (27.3%) |
| Disagree | 8 (26.7%) | 5 (26.3%) | 3 (27.3%) |
| No opinion | 10 (33.3%) | 6 (31.6%) | 4 (36.4%) |
| Agree | 3 (10.0%) | 2 (10.5%) | 1 (9.1%) |
| Strongly agree | 1 (3.3%) | 1 (5.3%) | 0 |

Responses to TFA question “*The Pause Grow Reflect programme has improved my self-care behaviour*”

| Rating | **All participants (n=30)**  n (%) | **Intervention group (n=19)**  n (%) | **Control group (n=11)**  n (%) |
| --- | --- | --- | --- |
| Strongly agree | 2 (6.7%) | 1 (5.3%) | 1 (9.1%) |
| Agree | 17 (56.7%) | 13 (68.4%) | 4 (36.4%) |
| No opinion | 8 (26.7%) | 3 (15.8%) | 5 (45.5%) |
| Disagree | 3 (10.0%) | 2 (10.5%) | 1 (9.1%) |
| Strongly disagree | 0 | 0 | 0 |

Responses to TFA question “*It is clear to me how the Pause Grow Reflect programme will help improve my self-care behaviour*”

| Rating | **All participants (n=30)**  n (%) | **Intervention group (n=19)**  n (%) | **Control group (n=11)**  n (%) |
| --- | --- | --- | --- |
| Strongly agree | 4 (13.3%) | 2 (10.5%) | 2 (27.2%) |
| Agree | 19 (63.3%) | 13 (68.4%) | 6 (54.5%) |
| No opinion | 5 (16.7%) | 2 (10.5%) | 3 (18.2%) |
| Disagree | 1 (3.3%) | 1 (5.3%) | 0 |
| Strongly disagree | 1 (3.3%) | 1 (5.3%) | 0 |

Responses to TFA question “*How confident did you feel about engaging with the Pause Grow Reflect programme?*”

| Rating | **All participants (n=30)**  n (%) | **Intervention group (n=19)**  n (%) | **Control group (n=11)**  n (%) |
| --- | --- | --- | --- |
| Very confident | 1 (3.3%) | 0 | 1 (9.1%) |
| Confident | 16 (53.3%) | 10 (52.6%) | 6 (54.5%) |
| No opinion | 3 (10.0%) | 2 (10.5%) | 1 (9.1%) |
| Unconfident | 8 (26.7%) | 6 (31.6%) | 2 (18.2%) |
| Very unconfident | 2 (6.7%) | 1 (5.3%) | 1 (9.1%) |

Responses to TFA question “*Engaging in the Pause Grow Reflect programme interfered with my other priorities”*

| Rating | **All participants (n=30)**  n (%) | **Intervention group (n=19)**  n (%) | **Control group (n=11)**  n (%) |
| --- | --- | --- | --- |
| Strongly disagree | 1 (3.3%) | 1 (5.3%) | 0 |
| Disagree | 12 (40.0%) | 8 (42.1%) | 4 (36.4%) |
| No opinion | 5 (16.7%) | 2 (10.5%) | 3 (27.3%) |
| Agree | 8 (26.7%) | 5 (26.3%) | 3 (27.3%) |
| Strongly agree | 4 (13.3%) | 3 (15.8%) | 1 (9.1%) |

Responses to TFA question “*How acceptable was the Pause Grow Reflect programme to you?”*

| Rating | **All participants (n=30)**  n (%) | **Intervention group (n=19)**  n (%) | **Control group (n=11)**  n (%) |
| --- | --- | --- | --- |
| Completely acceptable | 10 (33.3%) | 7 (36.8%) | 3 (27.3%) |
| Acceptable | 15 (50.0%) | 9 (47.4%) | 6 (54.5%) |
| No opinion | 3 (10.0%) | 1 (5.3%) | 2 (18.2%) |
| Unacceptable | 1 (3.3%) | 1 (5.3%) | 0 |
| Completely unacceptable | 1 (3.3%) | 1 (5.3%) | 0 |
